# Engrafting Horse Immune Cells into Mouse Hosts for the Study of the Acute Equine Immune Responses

**DOI:** 10.3390/ani11102962

**Published:** 2021-10-14

**Authors:** Caroline Leeth, Janie Adkins, Alayna Hay, Sophie Bogers, Ashley Potter, Sharon Witonsky, Jing Zhu

**Affiliations:** 1 Department of Animal and Poultry Sciences, 175 West Campus Drive MC 0306, Litton Reaves Hall rm 300, Blacksburg, VA 24061, USA; jea1026@vt.edu (J.A.); anw5137@vt.edu (A.H.); ashleyp8@vt.edu (A.P.); jing817@vt.edu (J.Z.); 2 Virginia Maryland College of Veterinary Medicine, 205 Duck Pond Drive, Blacksburg, VA 24061, USA; s0phie@vt.edu (S.B.); switonsk@vt.edu (S.W.)

**Keywords:** equine, NSG, xenograft, lymphocyte response

## Abstract

**Simple Summary:**

For decades, studies using research mice as models for disease have been critical to our current understanding of disease processes and associated immune responses, highlighting the ways in which mouse physiology is different from human and other species. Recent work has been directed at creating mice that can host human immune cells, allowing the study and manipulation of the human immune response without harm to patients. The purpose of this study was to explore to use of mouse hosts for horse immune cells. Horses are difficult to study immunologically as they are expensive to keep, and keeping their environment free of immune triggers is very difficult. Using mice allows us to increase our study numbers and control the environment which improves study reproducibility. In this study, we transferred both horse blood lymphocytes as well as horse bone marrow into specially modified mouse hosts. We found that mice are able to host horse immune cells and that these transferred cells are active. Future work can now build on this study to understand the horse immune response to infectious agents using mice, helping to identify new therapeutic tools to help equine patients.

**Abstract:**

Immunological studies in the horse are frequently hampered by lack of environmental control, complicated study design, and ethical concerns when performing high risk studies. The purpose of the current study was to investigate the utility of a xenograft model for studying acute equine immune responses. Immunocompromised non obese diabetic (NOD). sudden combined immunodeficiency (scid).gamma-/- (NSG) mice were engrafted with either equine peripheral blood lymphocytes (PBLs) or equine bone marrow to determine an optimal protocol for equine lymphocyte engraftment. We found that both PBL and bone marrow grafts populated the host mice successfully. Bone marrow transplants were technically more challenging and required further processing to retard graft versus host disease. Graft vs host disease was apparent at 28 days post-PBL transfer and 56 days post-bone marrow transfer. The results of these studies support the use of mouse hosts to study acute equine immune responses and that different engraftment techniques can be used depending on the experimental design.

## 1. Introduction

Equine immunological studies are frequently plagued by small sample populations, diverse patient populations, and the limited ability to control the research environment. Efforts to improve these limitations using equine subjects invariably results in exorbitant experimental costs as well as potentially raising ethical concerns. Compounding the problem, mammalian immune systems are species-specific and work carried out in human or other species may not readily translate to the horse. Therefore, basic research must be performed on the equine immune response to determine its unique profile. Only through this specific knowledge can we hope to improve health and novel therapeutic development for the equine population. 

Over the past several decades, the xenograft field has made great strides, successfully engrafting mouse hosts with tissues from other species. Much of this work has focused on engrafting human tumors as well as human immune cells. Success has been shown by engrafting other species as well including the dog, cow, and cat [1,2,3,4]. Work dating back to 1993 shows that equine peripheral blood leukocytes retain limited viability in a SCID/beige mouse host [5]. Over the several decades between then and now, the xenograft field has matured greatly. The NOD.Cg-*Prkdc^scid^Il2rg^tm1Wjl^*/SzJ (NOD.scid.gamma or NSG) has emerged as the premiere mouse model for this work, developed by Dr. Lenny Shultz of the Jackson Laboratory [6]. The NSG mouse is uniquely immunocompromised in that the NOD background has significantly dysfunctional natural killer cells, macrophages and dendritic cells. The severe combined immune deficiency (SCID) mutation blocks the development of lymphocytes and the interleukin 2 receptor deletion results in a lack of signaling from six major cytokines [7]. The NSG mouse hosts the highest percentage of human peripheral blood lymphocyte (PBL) engraftment compared to other models. Recently, this model was used to successfully engraft canine PBLs, achieving results similar to human xenografts [1]. Mice engrafted with human immune cells have been used to study infectious diseases, such as HIV, salmonella, and hepatitis C, as well as the autoimmune disorder, type 1 diabetes [8,9,10,11,12,13]. Pursuing the mouse to study the equine immune response offers an economical solution for increasing subject numbers as well as vastly improving our ability to control the experimental environment. All of these factors are critical to successful immunological research.

The purpose of this study was to investigate the use of the NSG mouse as a host for equine immune cells. Different methodologies were explored as well as initial explorations into equine immune cell responses post-engraftment. Our findings provide evidence that the use of mouse hosts for equine immune cell studies is a possible approach for future research. These preliminary results indicate that this methodology is worthy of pursuit with broad applications to improve clinical outcomes for equine patients.

## 2. Materials and Methods

### 2.1. Mice

NSG mice (JR 005557) were obtained from the Jackson Laboratory. All mice were housed in accordance with and procedures were performed under the guidance of Virginia Tech’s Animal Care and Use Committee (approval protocol 18-093). Mice were housed with medicated water containing sulfamethoxazole at 5 mg/kg over a 24-hour period [14] to limit nosocomial infections due to their extreme immunocompromised state. All mice were males between the ages of 8–12 weeks of age. 

### 2.2. Peripheral Blood Mononuclear Cell Engraftment

**Figure 1:**
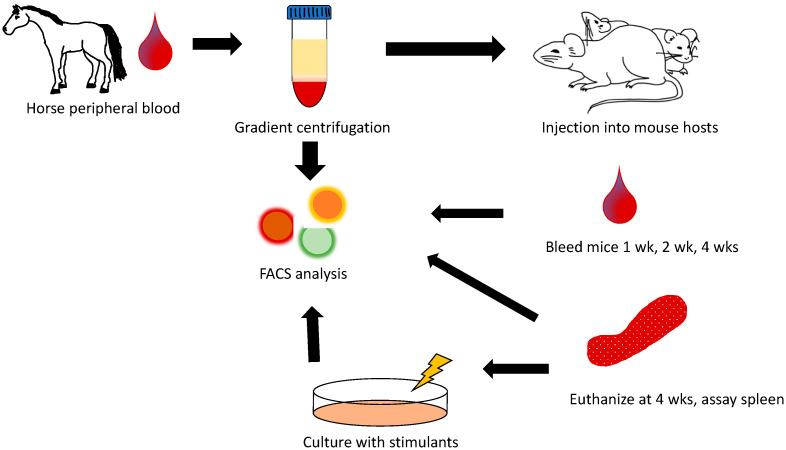
Diagram of experimental design for equine peripheral blood lymphocyte transfer into NSG mouse recipients. On day 0, equine peripheral blood was harvested from three donors. Gradient was centrifuged to separate out lymphocytes, counted and characterized for lymphocyte subsets, and injected IP at 5–10 × 10^6^ cells into each of 2 NSG hosts per donor horse. Mice were followed at day 7, 14, and 28 via peripheral blood analysis and sacrificed at 28 days. Splenic cellular analysis was then performed as well as cell culture with phorbol myristate acetate (PMA)/ionomycin stimulation. Cultured cells were analyzed on day 30 via flow cytometry. Wks = weeks, FACS = fluorescence activated cell sorting or flow cytometry.

Peripheral blood was collected from equine donors via jugular puncture using a 21-g vacutainer needle and heparinized vacutainer tubes. Donors were warmblood and thoroughbred geldings (*n* = 3, age 8–14 yrs), and 5 mL of blood was collected from each donor. PBLs were isolated using gradient centrifugation following manufacturer’s guidelines (Lympholyte, CedarLane Inc). Briefly, whole blood was diluted in 1 × PBS and then layered with Lympholyte. Cells were then centrifuged at 500G for 20 mins. Plasma was removed using sterile transfer pipettes and then the white blood cell layer was collected into clean conical tubes. Cells were washed twice with 1 × PBS. The supernatant was poured off, and cells were resuspended in sterile 1x PBS and counted (Cellometer, Nexcelom Bioscience, Lawrence, MA, USA). Cells were assessed via flow cytometry for lymphocyte percentages. Cells were then injected intraperitoneal into NSG recipient mice at 5–10 × 10^6^ cells per mouse. Two mice were engrafted per donor horse for a total of six mice (Figure 1). 

### 2.3. Bone Marrow Engraftment

**Figure 2:**
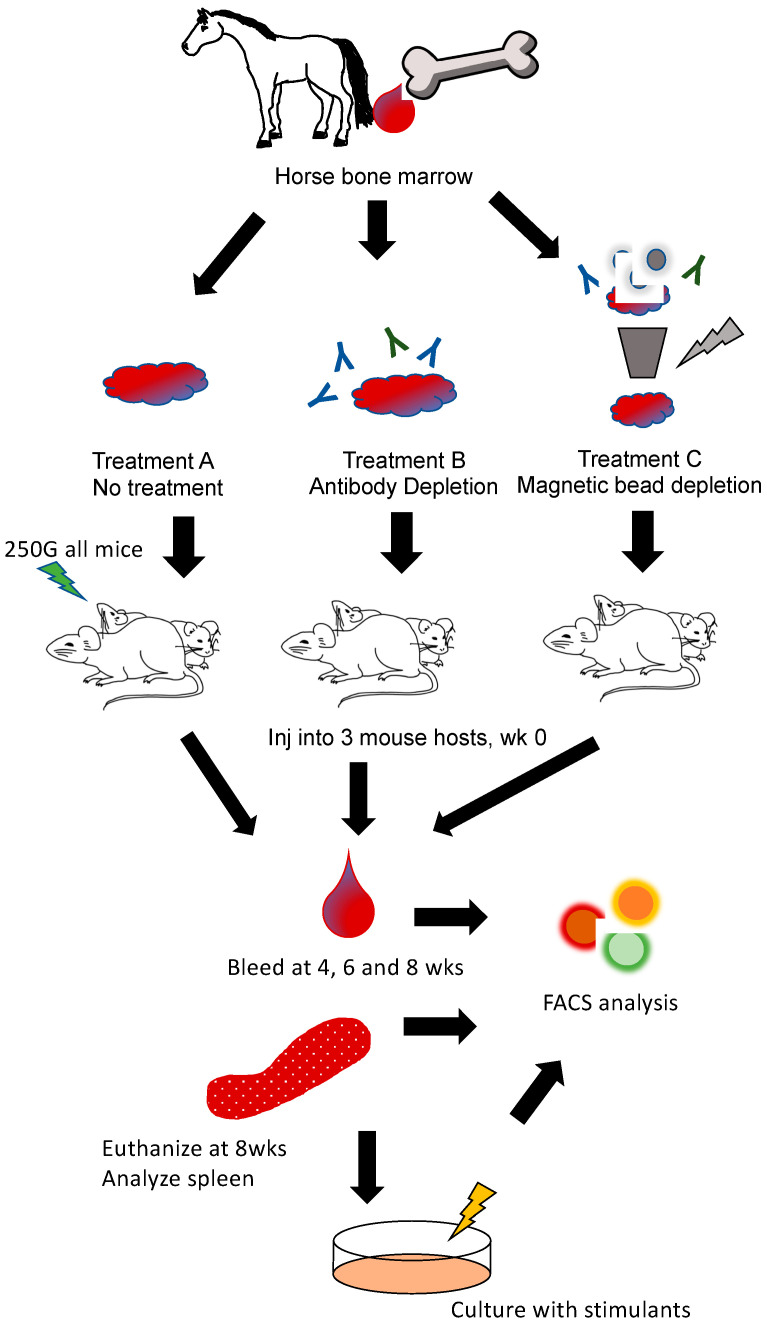
Diagram of experimental design for equine bone marrow cell transfer into NSG mouse recipients. On day 0, equine bone marrow was harvested from two donor horses. Bone marrow aliquots were either left untreated (treatment A), incubated with anti-equine T cell antibodies to deplete T cells (treatment B), or underwent magnetic bead depletion of equine T cells (treatment C). Cells (5–10 × 10^6^ via IP injection) were then transferred to 3 recipient mouse hosts. Peripheral blood samples were analyzed on day 28, day 42, and day 56. On day 56, mice were sacrificed and splenic cellular analysis was then performed as well as cell culture with LPS, ConA, and PWM stimulation. Cultured cells were analyzed on day 58 via flow cytometry. Inj = inject, Wks = weeks, FACS = fluorescence activated cell sorting or flow cytomtery.

Bone marrow was harvested from two equine donors (thoroughbred geldings ages 8–10 years). Horses were sedated with detomidine hydrochloride (0.01 mg/kg bwt). The area of the sternum was clipped and aseptically prepared. Local anesthetic (1mL mepivacaine) was placed in the subcutaneous tissues on midline over the 5th sternebrae. An 11-gauge, 10-cm Jamshidi needle was advanced through a stab incision into the sternebrae, and 15 mL of bone marrow was aspirated into 30-mL syringes which were pre-loaded with 5 mL of sodium heparin 1000 IU/mL (final concentration 250 IU/mL). Horses were administered with flunixin meglumine (1.1 mg/kg bwt) following bone marrow aspiration. The first aspirate syringe was transferred to a 60-mL conical tube and was used for engraftment. Bone marrow was treated with an Ammonium-Chloride-Potassium (ACK) lysis buffer and washed with Hanks balanced salt solution (HBSS). Cells were then separated into three different post-collection treatment groups. Treatment A cells were left unmanipulated. Treatment B cells were incubated for 30 mins with mouse anti-horse CD8 (clone CVS21, Bio-Rad), and mouse anti-horse CD4 (clone CVS4, Bio-Rad), at 1:1000 dilution, was washed and resuspended. Treatment C cells were incubated with mouse anti-horse CD4 (clone CVS4, BioRad), and mouse anti-horse CD8 (clone CVS21, BioRad), at 1:1000 dilution for 30 mins, was washed and then incubated with anti-mouse IgG microbeads (cat 130-048-401, Miltenyi Biotech) for 15 mins on ice. Cells were then run through LD columns per manufacturer’s directions (cat 130-042-901, Miltenyi Biotech, North Rhine-Westphalia, Germany). All recipient mice were irradiated with 250 gry 4 hours prior to cell transfer (RS 2000, Rad Source Technologies, Buford, GA, USA). For each of the three different cellular treatments, 2 mice were injected with 5 × 10^6^ cells IP per each equine donor (six mice total) (Figure 2).

### 2.4. Assessment of Engraftment

Equine PBLs were assessed for circulating lymphocyte percentages after gradient cell separation prior to injection. Briefly, 100 uL of equine donor blood was lysed with ACK buffer, and was washed and incubated at 4 degrees C for 30 minutes with the following antibodies: anti-horse CD4 (MCA1078F, Bio-Rad Laboratories, Hercules, CA, USA), anti-horse CD8 (MCA1080PE, BioRad), and CD21 (clone Bu33, Novus Biologicals, Littleron, CO, USA). Cells were then washed and stained with propidium iodide (PI, 421301, Biolegend, San Diego, CA, USA) for viability. After injection with equine PBLs, mice were bled after 1 week, 2 weeks, and 4 weeks to assess peripheral populations equine PBLs. Blood was collected in heparinized tubes and lysed using an ACK lysis buffer [15]. Cells were washed in FASC buffer and then centrifuged. Cells were stained with anti-horse CD4 (MCA1078F, BioRad), CD8 (MCA1080PE, BioRad), and CD21 (clone Bu33, Novus Biologicals). At 4 weeks post-PBL engraftment, mice were euthanized using CO_2_ asphyxiation followed by cervical dislocation in accordance with the regulatory guidelines [16]. Splenocytes were dissociated into a single cell suspension and red blood cells were lysed [17] (REF). Cells were resuspended at 5 × 10^6^/mL and assessed via flow cytometry for equine lymphocyte percentages using anti-horse CD4 (MCA1078F, BioRad), CD8 (MCA1080PE, BioRad), and CD21 (clone Bu33, Novus Biologicals). Mice from bone marrow transfer experiment were cheek bled at 4, 6, and 8 weeks post-engraftment and assessed via flow cytometry, as described above. Bone marrow transfer mice were sacrificed at 8 weeks post-engraftment and splenic analysis was performed as above. All cells were collected on an Attune NXT Cytometer (Life Technologies, Carlsbad, CA, USA) and data were analyzed using FlowJo software (FlowJo, LLC, Ashland, OR, USA).

### 2.5. Cell Culture

Splenocytes were harvested from mice after euthanasia. Euthanasia was performed using CO_2_ asphyxiation followed by cervical dislocation in accordance with the regulatory guidelines [16]. For the peripheral blood engrafted mice, cells were resuspended at 1 × 10^6^/mL and incubated with Cell Stimulation Cocktail (00-4975093, eBioscience) per manufacturer’s recommendations. After 48 hours, cells were harvested and assessed via flow cytometry for the presence of interferon gamma using anti-equine interferon gamma A647 antibody (clone 38-1, Cornell University). For the bone marrow engrafted mice, cells were stained with carboxyfluorescein succinimidyl ester (CFSE) (Thermo Fisher Scientific, Waltham, MA, USA), as previously described [18]. Cells were suspended at 1 × 10^6^/mL and plated with 5 uL/mL of lipopolysaccharide (LPS, 055:B5, Millipore Sigma, Burlington, MA, USA), 5 uL/mL of concanavalin A (ConA, 00497893, Invitrogen), or 2 ug/mL of pokeweed mitogen (PWM, L8777, Millipore Sigma). Cells were harvested after 48 hours and assessed for CSFE intensity as well as interferon gamma production (clone 38-1, Cornell University) using an Attune NXT Cytometer (Life Technologies) and FlowJo software (FlowJo, LLC).

### 2.6. Statistics

All results were assessed with two-way analysis of variance (ANOVA) using Prism software (version 9.1.2, Graphpad Software, LLC). 

## 3. Results

### 3.1. Equine Peripheral Blood Leukocytes Persist in NSG Recipient Mice up to 4 Weeks Post-Transfer

**Figure 3:**
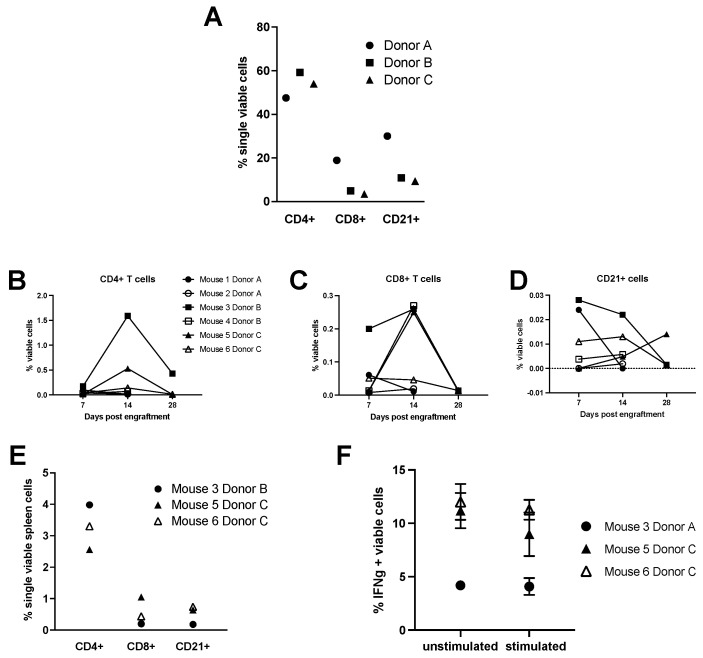
Equine PBL transfer into NSG hosts. (**A**) Donor horse peripheral blood lymphocyte populations post-gradient centrifugation separation and prior to engraftment. (**B**–**D**) Percentages of equine CD4+, CD8+, CD21+ lymphocyte populations, respectively, in the peripheral blood of mouse recipients at day 7, 14, and 28 post-engraftment. (**E**) Percentages of equine CD4+, CD8+, CD21+ lymphocyte populations in the spleens of mouse recipients at 28 days post-engraftment. Splenic cells from the remaining 3 mouse hosts were cultured for 12 hours in the presence of PMA and ionomycin. Interferon gamma positive cells were assessed and compared with unstimulated cells (**F**).

NSG recipient mice engrafted with equine peripheral blood leukocytes were followed for four weeks with two mice engrafted for each of three equine donors (Figure 1). Pre-engraftment percentages of peripheral B and T lymphocytes after gradient centrifugation separation showed no significant differences amongst the three donor horses (Figure 3A). CD4+ and CD8+ T lymphocytes as well as B lymphocytes were followed in the periphery for each mouse recipient after 1 week, 2 weeks, and 4 weeks of engraftment with no single donor outperforming another (Figure 3B–D). Mice one, two, and four perished prior to final analysis after 4 weeks. The cause of death was consistent with graft vs host disease including weight loss, ruffled hair coat, and lethargy [19]. Splenic lymphocyte populations were assessed in the remaining three mice 4 weeks post-transfer and no significant differences were found (Figure 3E). To assess the functional capacity of the transferred equine lymphocytes, splenic cells from the remaining mice surviving at 28 days post-transfer were cultured with Cell Stimulation Cocktail which contains PMA, ionomycin, brofeldin A, and monensin. After 12 hours of culture, cells were assessed for interferon gamma production using flow cytometry, and all wells, regardless of stimulation, were found to produce similar amounts of interferon gamma (Figure 3F). 

### 3.2. Engraftment of Equine Bone Marrow into NSG Mice Is Variable Depending on Transplantation Technique

**Figure 4:**
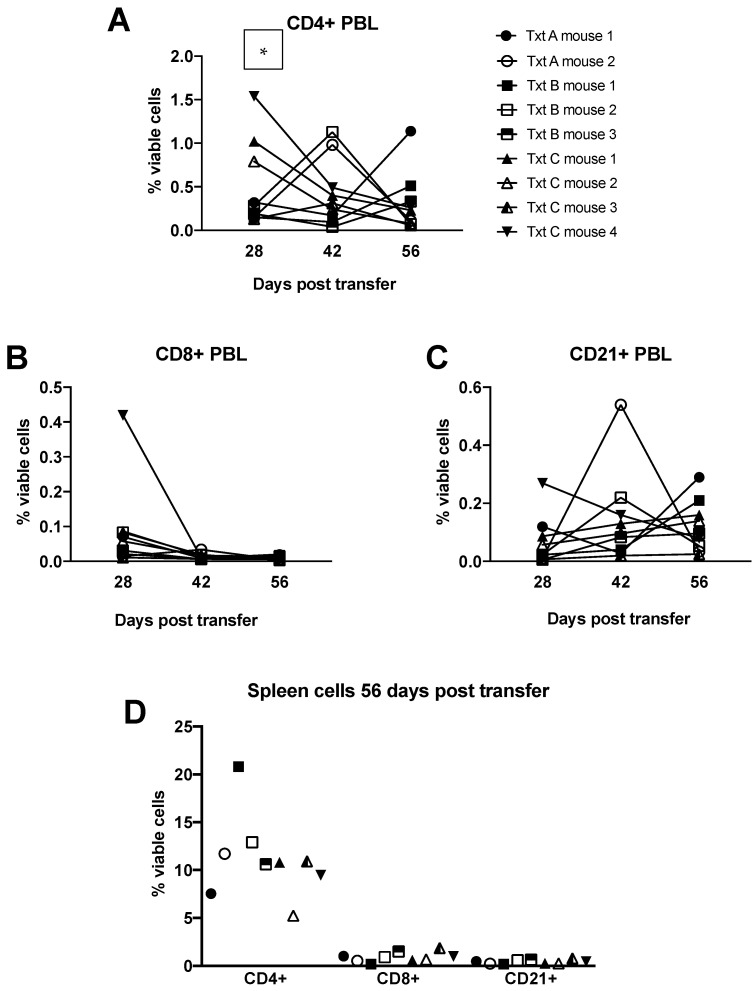
Equine bone marrow transfer into NSG hosts. (**A**–**C**) Percentages of equine CD4+, CD8+, CD21+ lymphocyte populations, respectively, in the peripheral blood of mouse recipients at day 28, 42 and 56 post-engraftment. (**D**) Percentages of equine CD4+, CD8+, CD21+ lymphocyte populations in the spleens of mouse recipients at 56 days post-engraftment. Txt = treatment. * *p* = 0.0285 using a paired, two-tailed *t*-test.

**Figure 5:**
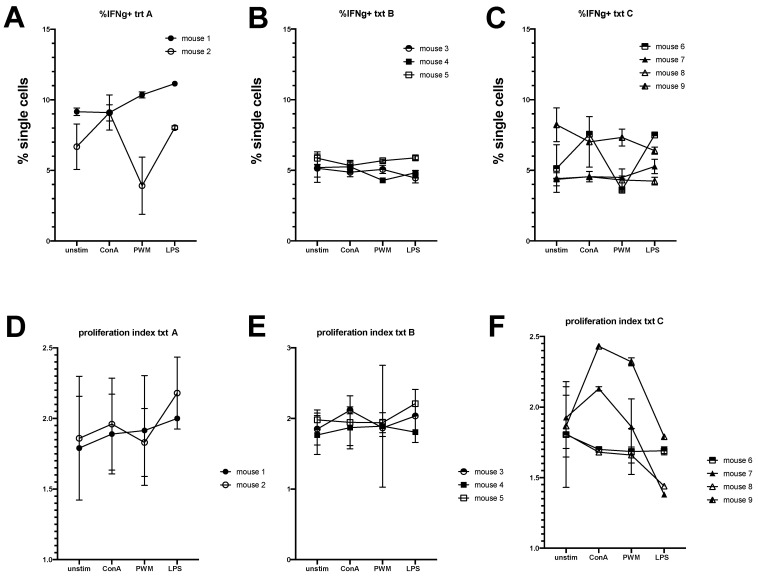
Assessment of functional capacity of transferred equine bone marrow cells retrieved from host spleens 56 days post-engraftment. Splenic cells were labelled with CFSE and cultured for 48 hours under different stimulants—ConA, PWM, and LPS. (**A**–**C**) Interferon-positive cell percentages were assessed for the three different treatment groups. (**D**–**F**) Proliferation index based on CFSE fluorescence was assessed for the three different treatment groups.

Bone marrow was extracted from the sternum from each donor horse and engrafted into irradiated recipient NSG mice. Three treatment schemes were assessed: treatment A (no manipulation), treatment B (depletion of T lymphocytes using magnetic bead separation), and treatment C (incubation with anti-horse T cell antibodies to deplete T cells). Blood samples collected at 28, 42, and 56 days post-engraftment showed no differences in circulating lymphocytes among the different treatment groups except at day 28 where treatment group C had significantly more CD4+ T cells than treatment group A (Figure 4A–C). One mouse from treatment B group and two mice from treatment A group died prematurely prior to the first assessment with cause of death being undetermined. Splenic lymphocyte populations were assessed at 8 weeks post-transfer and no differences were found in lymphocyte percentages among the remaining mice (Figure 4D). Splenic lymphocytes were cultured with LPS, ConA, or PWM and stained with CFSE for proliferation evaluation. No differences in the expression of interferon gamma to culture stimulation for the different treatment groups were found (Figure 5A–C), and the proliferation index (function of Flowjo software, version 10.7.1, Ashland, OR, USA) for each group did not differ significantly (Figure 5 D–F). 

## 4. Discussion

Xenografts using mouse hosts offer improved environmental control, replication ability, and, thus, greater reproducibility in studying acute immune responses to pathogens in horses. While the human to mouse xenograft field is making significant strides, work on engrafting other species into the mouse has been slow. Admittedly, the cost of this work is not insignificant and may continue to be a hurdle; however, the expense of using larger species in research is also high. Many studies use the mouse as a host for xenographic tumor material, testing susceptibility of the cancer to therapeutics prior to trial in patients [20,21,22,23]. In this study, we investigated several different techniques of engraftment of equine lymphocytes into mouse hosts to determine the most appropriate model to study acute equine lymphocytic immune responses. 

Engraftment of equine peripheral blood lymphocytes was successful and circulating cells were detected in the mouse hosts. Our results are similar to those found after engrafting canine PBLs into NSG mouse hosts in which circulating cells were identified 9 days post-engraftment and graft versus host responses were seen at +28 days [1]. Greater availability of canine lymphocyte subset markers allowed more in-depth cell identification than the current repertoire of anti-equine antibodies for lymphocyte cell surface markers [1]. This canine manuscript irradiated the murine hosts; however, methods published using human PBLs did not require irradiation [24]. As consistent with other adaptive transfer techniques familiar to us [15,18], we did not irradiate mice for the PBL transfer experiments. A study transferring bovine PBLs into beige SCID (C.B-17 scid/scid) mice compared irradiation vs non-irradiation transfer techniques of PBLs. As these SCID mice are not as immunosuppressed as NSG mice, this study concluded that transferred cells engrafted better with irradiation; however, the evidence is not strong. Circulating bovine cells were found in this model at similar time points to the canine model and our equine model; however, no overt graft vs host disease was suspected [2]. A second study by this group found that the engrafted bovine PBLs retained some immune function; however, through a skin grafting method, it differed from the assessment used in this study [3]. Studies using human hematopoietic xenografts have found that further modification of the NSG mouse to include human transgenic cytokine expression (termed NSGS mice) created greater functional engraftment of functional cells, a leap not yet possible for equine xenograft experiments [25].

Variation exists among equine individuals in the percentage of CD4+ and CD8+ T cells and B cells in peripheral blood (Figure 3A) and should be taken into account when performing engraftment of these cells. Equine lymphocytes were found in circulation as well as populating the spleen of the mouse hosts at the earliest time points examined. Several mice were lost between day 14 and day 28 sampling time points. The cause of this loss was not able to be decerned on necropsy; however, nosocomial infections and/or graft vs host disease was suspected. A more broad-spectrum antibiotic therapy should be considered for future engraftments to help prevent nosocomial infections as NSG mice are highly immunosuppressed [26]. In addition, our results suggest that experimentation on/with the engrafted cells should be performed by 7 days post-transfer to ensure adequate time for study as well as sufficient host survival. These suggestions are supported by our 28-day post-transfer culture results in which equine cells were found to be producing interferon gamma, but not in response to the added stimulants (Figure 3A,B). Cells appear to respond regardless of treatment during culture, presumably due to activation from graft vs. host responses which typically appears after 3–4 weeks of PBL transfer [1,27]. 

Our equine bone marrow transfer results indicate that the grafts should receive some treatment for T-cell depletion prior to transfer (Figure 4). Reports show that circulating T-cells can be present in the bone marrow and contribute to graft versus host disease. The depletion of these cells increases the survival of the transplanted bone marrow cells in the recipients [19]. Bone marrow that received no treatment (treatment A) had poor host survival at 28 days post-transfer with only 2 of 4 mice surviving to the end of the study. Bone marrow transfer protocols typically recommend allowing cells to rest and engraft for 4 weeks before manipulating the mice. Depleting T lymphocytes from the PBL samples would result in no T lymphocyte engraftment; however, these cells will arise from lymphoid progenitor cells in the bone marrow transfer so depleting these circling T lymphocytes is advisable. Treatment with antibody to deplete T lymphocytes resulted in the loss of 1 of 4 recipient mice, and treatment that used magnetic beads to deplete T lymphocytes did not result in any early host mouse loss. All surviving mice showed similar engraftment of cells at the time points examined. Based on peripheral cell assessment, experimentation with engrafted mice can begin 28 days post-bone marrow transfer. This suggestion is supported by the stimulation data which showed that all cells in the mice were reactive regardless of culture stimulant used, indicating active graft vs host disease at 56 days post-engraftment (Figure 5). The development of graft versus host disease in the recipient mice also suggests that the engrafted cells are indeed responsive and should be active for experimentation.

## 5. Conclusions

Our results support the utility of a xenographic mouse model for the study of acute equine lymphocytic responses. Both the peripheral blood lymphocyte as well as the T-cell-depleted bone marrow transfers were successful in establishing functional lymphocyte populations in host mice. The xenograft model used would depend on the study to be conducted. More work is needed to refine this technique; however, these preliminary studies show great promise for these novel models. 

## Data Availability

No new data sets were created or analyzed in this study. Data sharing is not applicable to this article.
